# Supplementary material for: Gene model for the ortholog of Ilp4 in Drosophila eugracilis
Source: bioRxiv. 2025 Sep 8:2025.09.05.674564. Preprint. [Version 1] doi: 10.1101/2025.09.05.674564 (PMC12439880; doi:10.1101/2025.09.05.674564)
Supplement: Supplement 2 [file NIHPP2025.09.05.674564v1-supplement-2.pdf]

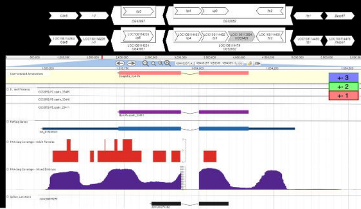

User-created Annotations
DeugGB2_Ilp4-PA
D. mel Proteins
RefSeq Genes
RNA-Seq Coverage - Adult Females
RNA-Seq Coverage - Mixed Embryos
Splice Junctions

C.

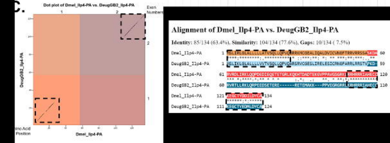

Dot plot of Dmel_Ilp4-PA vs. DeugGB2_Ilp4-PA
Exon Numbers
DeugGB2_Ilp4-PA
Dmel_Ilp4-PA
Amino Acid Position
Alignment of Dmel_Ilp4-PA vs. DeugGB2_Ilp4-PA
Identity: 85/134 (63.4%), Similarity: 104/134 (77.6%), Gaps: 10/134 (7.5%)
Dmel_Ilp4-PA
DeugGB2_Ilp4-PA
